# A single consumption of curry improved postprandial endothelial function in healthy male subjects: a randomized, controlled crossover trial

**DOI:** 10.1186/1475-2891-13-67

**Published:** 2014-06-28

**Authors:** Hideki Nakayama, Nobuaki Tsuge, Hiroshi Sawada, Noriya Masamura, Shohei Yamada, Shigeki Satomi, Yukihito Higashi

**Affiliations:** 1 Research & Development Institute, House Foods Corporation, Yotsukaido 284-0033, Japan; 2 Central Research & Development Institute, House Foods Group Inc., Yotsukaido 284-0033, Japan; 3 Department of Regeneration and Medicine, Research Institute for Radiation Biology and Medicine, Hiroshima University, Hiroshima 734-8553, Japan

**Keywords:** Curry, Spice, Antioxidant, Endothelial function, Postprandial hyperglycemia, Flow-mediated vasodilation, Prevention, Cardiovascular events

## Abstract

**Background:**

Curry, one of the most popular foods in Japan, contains spices that are rich in potentially antioxidative compounds, such as curcumin and eugenol. Oxidative stress is thought to impair endothelial function associated with atherosclerosis, a leading cause of cardiovascular events. The aim of this study was to determine whether a single consumption of curry meal would improve endothelial function in healthy men.

**Methods:**

Fourteen healthy male subjects (BMI 23.7 ± 2.7 kg/m^2^; age 45 ± 9 years) were given a single serving of curry meal or spice-free control meal (180 g of curry or control and 200 g of cooked rice; approximately 500 kcal in total) in a randomized, controlled crossover design. Before and 1 hr after the consumption, fasting and postprandial flow-mediated vasodilation (FMD) responses and other parameters were measured.

**Results:**

The consumption of the control meal decreased FMD from 5.8 ± 2.4% to 5.1 ± 2.3% (*P* = 0.039). On the other hand, the consumption of the curry meal increased FMD from 5.2 ± 2.5% to 6.6 ± 2.0% (*P* = 0.001), and the postprandial FMD after the curry meal was higher than that after the control meal (*P* = 0.002). Presence of spices in the curry did not alter significantly the systemic and forearm hemodynamics, or any biochemical parameters including oxidative stress markers measured.

**Conclusions:**

These findings suggest that the consumption of curry ameliorates postprandial endothelial function in healthy male subjects and may be beneficial for improving cardiovascular health.

**Trial registration:**

UMIN Clinical Trials Registry 000012012.

## Background

Curry originated in Indian traditional diet, and has become widely eaten throughout the world, especially in Asia [1]. In fact, curry is one of the most popular foods in Japan [1]. Japanese curry is often milder in flavor and thicker in consistency than the traditional Indian curry, and usually is served with cooked rice [1]. With a good amount of meat and vegetables in it, Japanese curry can make a convenient and nutritious meal for people of all ages [1].

While being mild in flavor, Japanese curry still contains an abundant amount of spices, some of which are high in antioxidants. For example, turmeric contains antioxidant yellow pigment, curcumin, which is known to have many health benefits such as vasoprotective, antiinflammatory, anticarcinogenic, and neuroprotective effects [2,3]. Clove contains antioxidant aromatic oil, eugenol, which is also known to have some health benefits such as vasoprotective and pulmonary protective effects [4,5]. Epidemiologic studies have shown that curry improves pulmonary function in Asian elderly adults [6] and curry consumption improved cognitive performance of nondemented elderly Asians [7]. However, few intervention studies about curry have been reported.

It is well known that postprandial hyperglycemia is a contributing factor to the development of atherosclerosis and is a risk factor for cardiovascular events. A meta-regression analysis showed that the progressive relationship between glucose levels and cardiovascular risk extends even in subjects with normal glucose tolerance below the diabetic threshold [8].

Although mechanism by which postprandial hyperglycemia induces vascular dysfunction is not fully understood, a review by Mar and Bruno points out that oxidative stress-mediated disruptions in nitric oxide homeostasis have been implicated as key events leading to vascular dysfunction [9]. Glucose loading produced a decrease in endothelial function and an increase in a marker of oxidative stress in normal and diabetic subjects [10,11]. Hyperglycemia in response to oral glucose loading rapidly suppressed endothelium-dependent vasodilation, probably through increased production of oxygen free radicals [11-13].

Accumulating evidence suggests that endothelial dysfunction plays a crucial role in the development and progression of atherosclerosis. The endothelium is suggested to be a target of damage in the postprandial state [14-16].

The aim of this study was to determine whether a single consumption of a dish of Japanese curry and rice would improve postprandial endothelial function in healthy men.

## Methods

### Subjects

In December 2012, 18 healthy males aged 33 to 64 years were recruited for the study. We selected healthy male subjects who do not have any history of hypertension, diabetes mellitus, and dyslipidemia, to avoid the possibility of any influence on the endothelial function by these factors as well as by menstrual cycle. The study protocol was approved by Tana Orthopedic Surgery Institutional Review Board. Informed consent for participation in the study was obtained from all subjects.

### Study design

The study was performed at House Foods Corporation during the period from January 2013 to February 2013. Subjects were randomized into a control-first group and a curry-first group. Each subject was given a single serving of test meal (control meal or curry meal) in a crossover manner with more than one week in between the two test meals. Fasting and postprandial measurements were taken by the primary investigator under blinded condition as follows.

Subjects were asked not to take any nutritional supplements on the day before the test and were fasted for at least 12 hours overnight prior to the fasting measurements. No other instructions were given to the subjects. After the fasting measurements, subjects were given 180 g of either control (198 kcal) or curry (187 kcal) and 200 g of cooked rice (294 kcal) by a support staff, and one hour afterwards, postprandial measurements were taken. A preliminary test which we had made before this study showed that a measurement one hour after the intake of the control meal showed low endothelial function in healthy subjects but this effect was not observed two hours after the intake of the control meal. For this reason, we thought that the single point measurement one hour after meal was appropriate for studying curry effects on postprandial endothelial dysfunction in healthy subjects.

The curry consisted of ground beef, tomato, tomato puree, some seasonings, and a blend of spices (Table 1). The control was prepared as the curry except that all the spices were removed from the formula; it tasted like a meat sauce for spaghetti. The nutritional compositions of the control meal and curry meal both with 200 g of cooked rice were essentially the same as shown in Table 2.

**Table 1:** Composition of the spice blend used in a single serving portion of the curry meal

**Spice**	**Amount**
Clove (g)	0.9
Coriander (g)	1.8
Cumin (g)	0.9
Garlic (g)	3.6
Ginger (g)	2.7
Onion (sautéed) (g)	9
Red pepper (g)	0.09
Turmeric (g)	4.5

**Table 2:** Composition of a single serving portion of the test meal with 200 g of rice

**Component**	**Control**	**Curry**
Energy (kcal)^*a*^	492	481
Protein (g)^*b*^	15	16
Fat (g)^*c*^	11	10
Carbohydrate (g)^*d*^	82	82
Dietary fiber (g)^*e*^	3	3
Sodium (g)^*f*^	1	1

^*a*^Modified Atwater methods.

^*b*^ Kjeldahl nitrogen determination method (conversion factor: 6.25).

^*c*^Ether extraction method.

^*d*^Carbohydrate was calculated from protein, fat, ash (direct ashing), and moisture (oven drying at 105°C for 16 hours).

^*e*^Modified Prosky method.

^*f*^Inductively coupled plasma emission spectrometry.

Single serving portions (180 g) of both the curry and the control were packed and sterilized in retort pouches and were stored at room temperature until use. They were heated in boiling water for five minutes just before serving with 200 g of cooked rice.

### FMD and hemodynamic measurements

All measurements were taken in the morning, in a quiet, air-conditioned room maintained at 24°C and 50% relative humidity. The subjects rested quietly in the supine position for 20 min before and during each FMD measurement. Just before the cuff inflation, systolic and diastolic blood pressure was measured on the left arm.

FMD was measured on the right arm as described previously [17] with a UNEXEF18G ultrasound imaging system programmed specifically for FMD measurements with the autocuff (UP310), the semi-automatic vessel tracking (X-link), and the flow mode (uFFLOW) options (UNEX Co., Nagoya, Japan). After the brachial artery diameter was determined for the baseline arterial diameter from a longitudinal image of the artery acquired 5 to 10 cm above elbow, reactive hyperemia was induced by 5 min of distal lower arm occlusion by a blood pressure cuff inflated to 50 mm Hg above systolic pressure. Subsequently, the longitudinal image of the artery was recorded continuously until 2 min after the cuff deflation for the maximal brachial artery diameter determination. Pulsed Doppler velocity signals were acquired for 20 seconds before cuff inflation and for 10 seconds after cuff deflation for the hyperemic flow (% increase in blood flow) determination.

We have already estimated the reproducibility and accuracy of the measurement by a preliminary test using 10 subjects. The same person, who performed the FMD measurement of this study, had measured FMD of each subject twice at one hour interval. The interclass correlation coefficient was 0.98 and systematic error across these two measurements was 0.1%.

### Serum measurements

Whole blood samples were collected from the left arm, just after the FMD and hemodynamic measurements. They were separated immediately by centrifugation (1,200 g, 10 min) and were stored at -80°C until use. Glucose, triglycerides, and total cholesterol were measured by enzymatic procedures (Mitsubishi Chemical Medience Corporation, Tokyo, Japan). High-density lipoprotein cholesterol (HDL-C) and low-density lipoprotein cholesterol (LDL-C) were measured by enzymatic procedures (Kyowa Medex Co., Ltd., Tokyo, Japan). Insulin was measured by Chemiluminescent immunoassay (ABBOTT JAPAN Co., Ltd, Tokyo, Japan). High-sensitivity C-reactive protein (hs-CRP) was measured by nephelometry using N-latex CRP-2 (Siemens Healthcare Japan, Tokyo, Japan). Malondialdehyde-modified LDL (MDA-LDL) was measured by two step ELISA (SEKISUI MEDICAL Co., Ltd., Tokyo, Japan) which uses monoclonal antibody recognizing MDA residues (ML25) and monoclonal antibody against apoB (AB16) to make the assay specific to MDA-LDL as described previously [18].

### Urine measurement

For the urine 8-isoprostane determinations, a single voided urine sample was collected from each subject just after the blood sampling and was stored at -80°C until use. To 1000 μl (Creatinine < 50 mg/dl) or 500 μl (Creatinine ≥ 50 mg/dl) urine sample, 3H-8epi PGF2α (20 dpm/μL) 50 μL and 4% acetic acid 3 ml were added. After being stirred for 30 min, the sample was applied on an OASIS HLB solid phase extraction column (Nihon Waters K.K., Tokyo, Japan) preconditioned with 2 ml each of Ethylacetate and Acetonitryl and 3 ml of 4% acetic acid). After being washed with 15% acetonitry in Ehylacetate: hexane: 1-butanol = 15: 85: 1, the column was eluted with 2 ml ethylacetate:hexane = 85: 1. The eluted sample was dried under nitrogen, dissolved in 1 ml EIA buffer, and stirred for 30 min before being subjected to EIA assay (Cayman Chemical, Michigan, United States) as described previously [19]. The urine creatinine for normalization of 8-isoprostane concentration was measured by enzymatic procedures (Mitsubishi Chemical Medience Corporation, Tokyo, Japan).

### Statistical analysis

A power analysis for two-way within subjects ANOVA was performed as described [20] and showed that a total sample size of 12 subjects was required to detect an interaction between meals and prandial status with an α error of 0.05 and a power of 0.80. Data are presented as means ± standard deviations. Baseline characteristics were compared between the curry-first and the control-first group by unpaired *t*-test. Statistical analyses about the effects of curry on FMD and other parameters were performed by using two-way within subjects ANOVA (control/curry and fasting/postprandial). If the interaction was significant, post hoc Student’s paired *t*-test was performed. Statistical significance was assumed if a null hypothesis could be rejected at *P* = 0.05. All analyses were performed with SPSS (version 22; IBM Japan, Ltd., Tokyo, Japan).

## Results

### Baseline characteristics

Of the 18 subjects enrolled in the study, 4 subjects were excluded from the analysis for the follwoing reasons. One subject canceled after the first participation. The ultrasound images of another subject were technically unsuitable for the analysis, Another subjects had a sleeping problem on the night before the test day, and the other subject showed very low postprandial glucose level (<50 mg/dL). Table 3 summarizes the baseline characteristics of the remaining 14 subjects. All baseline characteristics did not differ significantly between the control-first and the curry-first group.

**Table 3:** Baseline characteristics

**Parameters**	**Total (n = 14)**	**Control-first (n = 7)**	**Curry-first (n = 7)**
Age (years)	45 ± 9	42 ± 7	48 ± 10
Body mass index (kg/m^2^)	23.7 ± 2.7	23.0 ± 1.9	24.4 ± 3.3
Systolic blood pressure (mm Hg)	112 ± 8	112 ± 5	112 ± 11
Diastolic blood pressure (mm Hg)	75 ± 7	76 ± 5	74 ± 9
Heart rate (beats/min)	60 ± 10	59 ± 7	61 ± 13
Total cholesterol (mg/dL)	196 ± 34	194 ± 34	199 ± 37
Triglycerides (mg/dL)	97 ± 44	99 ± 53	96 ± 38
HDL-cholesterol (mg/dL)	58 ± 15	58 ± 14	57 ± 17
LDL-cholesterol (mg/dL)	118 ± 28	114 ± 29	121 ± 28
Glucose (mg/dL)	90 ± 7	88 ± 6	91 ± 8
Insulin (μU/mL)	4.1 ± 1.9	3.9 ± 1.5	4.3 ± 2.4
hs-CRP (mg/dL)	0.122 ± 0.179	0.094 ± 0.176	0.149 ± 0.192
MDA-LDL (U/L)	88 ± 20	83 ± 20	93 ± 21
8-isoprostane (pg/mg Cr)	285 ± 81	276 ± 95	294 ± 70

Values are means ± SD.

*Abbreviations*: *HDL* high-density lipoprotein, *LDL* low-density lipoprotein, *hs-CRP* high-sensitivity C-reactive protein, *MDA* malondialdehyde-modified, *Cr* creatinine.

### Effects of curry consumption on FMD and other hemodynamic parameters

Table 4 summarizes the brachial and hemodynamic parameters with significance probability values (*P* values) from the ANOVA results.

**Table 4:** Vascular function and Systemic Hemodynamics

	**Control**		**Curry**			***P *** **values**^***a***^	
**Parameters**	**Fasting**	**Postprandial**	**Fasting**	**Postprandial**	**Meal effects**	**Interaction**	**Prandial effects**
FMD (%)	5.8 ± 2.4	5.1 ± 2.3	5.2 ± 2.5	6.6 ± 2.0	0.20	< 0.001	0.25
Systolic blood pressure (mm Hg)	110 ± 7	110 ± 10	111 ± 8	113 ± 7	0.07	0.57	0.33
Diastolic blood pressure (mm Hg)	73 ± 8	67 ± 7	75 ± 7	70 ± 7	0.07	0.32	< 0.001
Heart rate (beats/min)	60 ± 10	66 ± 11	59 ± 9	66 ± 10	0.56	0.55	< 0.001
Baseline arterial diameter (mm)	4.00 ± 0.47	4.12 ± 0.49	3.96 ± 0.47	4.02 ± 0.46	0.12	0.07	< 0.001
Peak hyperemic flow (%)	595 ± 259	536 ± 157	609 ± 217	571 ± 170	0.60	0.76	0.22

Values are means ± SD.

*Abbreviations*: *FMD* flow-mediated vasodilation.

^*a*^*P* values were calculated by using two-way within subjects ANOVA (control/curry and fasting/postprandial).

**Figure 1:**
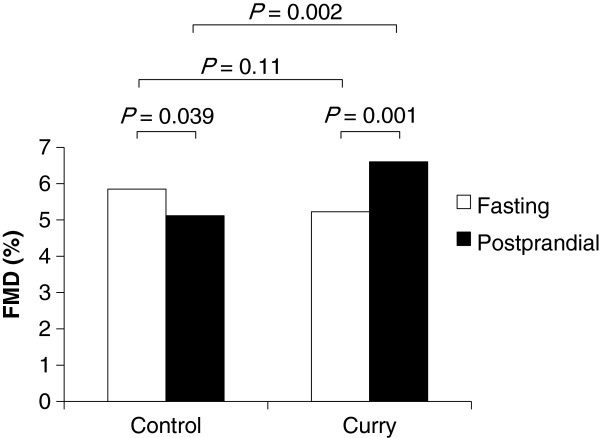
**Effects of a single consumption of curry on flow-mediated vasodilation (FMD), n = 14.** Because the interaction between meals (control/curry) and prandial status (fasting/postprandial) was found significant (*P* = 0.002) by using two-way within subjects ANOVA, *P* values were calculated by post hoc Student’s paired *t*-test. Abbreviations: *FMD* flow-mediated vasodilation.

Only on FMD, the interaction effect between meals (control/curry) and prandial status (fasting/postprandial) was significant (*P* < 0.001). Post hoc Student’s paired *t*-tests revealed that the consumption of the control significantly decreased the FMD from 5.8 ± 2.4% to 5.1 ± 2.3% (Figure 1, *P* = 0.039). On the other hand, the consumption of the curry increased FMD from 5.2 ± 2.5% to 6.6 ± 2.0% (*P* = 0.001), and the postprandial FMD after the curry meal was higher than that after the control meal (5.1 ± 2.3% vs. 6.6 ± 2.0%, *P* = 0.002).

After consumption of the test meals, heart rate and baseline arterial diameter increased significantly, and diastolic blood pressure decreased significantly. Other than these, no main effects of the meals, the prandial status, or no interaction effects on systemic and forearm hemodynamics were significant.

### Effects of curry consumption on biochemical parameters

Table 5 summarizes the biochemical parameters with *P* values from the ANOVA results.

**Table 5:** Biochemical parameters

	**Control**		**Curry**			***P *** **values**^***a***^	
**Parameters**	**Fasting**	**Postprandial**	**Fasting**	**Postprandial**	**Meal effects**	**Interaction**	**Prandial effects**
Total cholesterol (mg/dL)	203 ± 36	195 ± 34	197 ± 32	191 ± 28	0.40	0.36	0.001
Triglycerides (mg/dL)	107 ± 59	114 ± 59	97 ± 44	103 ± 43	0.23	0.96	0.025
HDL-cholesterol (mg/dL)	58 ± 16	56 ± 16	59 ± 15	56 ± 15	0.55	0.53	< 0.001
LDL-cholesterol (mg/dL)	122 ± 30	118 ± 28	118 ± 27	112 ± 23	0.36	0.42	0.001
Glucose (mg/dL)	89 ± 7	113 ± 24	90 ± 7	117 ± 29	0.25	0.44	0.001
Insulin (μU/mL)	3.9 ± 2.0	26.0 ± 11.7	4.3 ± 1.9	25.5 ± 9.9	0.98	0.68	< 0.001
hs-CRP (mg/dL)	0.107 ± 0.159	0.104 ± 0.158	0.089 ± 0.145	0.087 ± 0.143	0.66	0.87	0.038
MDA-LDL (U/L)	94 ± 24	85 ± 24	95 ± 26	85 ± 21	0.93	0.91	0.027
8-isoprostane (pg/mg Cr)	273 ± 76	283 ± 89	277 ± 69	324 ± 67	0.23	0.17	0.047

Values are means ± SD.

*Abbreviations*: *HDL* high-density lipoprotein, *LDL* low-density lipoprotein, *hs-CRP* high-sensitivity C-reactive protein, *MDA* malondialdehyde-modified, *Cr* creatinine.

^*a*^*P* values were calculated by using two-way within subjects ANOVA (control/curry and fasting/postprandial).

While the main effects of meals and the interaction effects between meals (control/curry) and prandial status (fasting/postprandial) were not significant, the main effects of prandial status were significant for all parameters measured.

## Discussion

In the present study, we demonstrated that a single consumption of a dish of curry and rice improved the postprandial FMD in healthy men.

We believe the significant interaction effect on FMD between meals and prandial status was due to the antioxidant components in the curry, although consumption of the curry meal did not change the two oxidative stress parameters (MDA-LDL and urine 8-isoprostane) measured in our study. Many researchers reported that FMD was improved by some antioxidant consumption (long term and short term) without 8-isoprostane decrease [21-23]. Few intervention studies using antioxidants report serum MDA-LDL levels. Pfeuffer et al. reported that consumption of 150 mg/d quercetin for 8 weeks affected neither oxidized LDL nor 8-isoprostane [24]. These reports suggest that no change in MDA-LDL and urine 8-isoprostane does not necessarily mean that improvement in FMD is not attributable to decrease in oxidative stress. On the other hand, it has been reported that administration of glucose in oral glucose tolerance test (OGTT) impaired endothelial function with concomitant increase in postprandial oxidative stress [11]. It is also reported that the postprandial serum glucose after rice is lower than that after the equivalent amount of glucose consumption [25]. In our study, we found that the test meals containing 82 g carbohydrate, which is nearly equivalent to the amount of glucose administered in 75-g OGTT, increased postprandial serum glucose and a consumption of the control meal resulted in decrease in FMD suggesting impairment of endothelial function. We speculate that the presence of spice antioxidants in the curry would have prevented the increase in the oxidative stress induced by postprandial serum glucose increase, but the change in the oxidative stress parameters after our test meal was too small to be detected, because the postprandial serum glucose level after our test meals (113 mg/dL after control or 117 mg/dL after curry) was much lower than that normally encountered after 75-g oral glucose loading [26].

Curcumin, eugenol and quercetin in curry could have contributed to our results suggesting that spices improved postprandial endothelial function. It was reported that curcumin alleviates an endothelium-dependent vasodilator dysfunction induced by high glucose in rat aortic rings and increased heme oxygenase-1 activity, and that stimulation of guanylyl cyclase may be involved in the protective effects of curcumin [27]. Eugenol was reported to produce smooth muscle relaxation resulting from the blockade of both voltage-sensitive and receptor-operated channels that are modulated by endothelial-generated nitric oxide [4]. It has been also suggested that qurcetin, which is a main polyphenol of onion and is also contained in curry, is known as a selective modulator of cyclic GMP-dependent relaxations [28]. Future studies are needed for elucidating the mechanism of our findings.

After consumption of the test meals, baseline arterial diameter increased significantly. Similar increase in baseline arterial diameter after a fatty meal consumption has been reported [29,30]. Although many researchers reported that increase in baseline diameter would decrease FMD [31,32], the increase did not appear to have diminished the effect of spices on FMD, because significant interaction effect between meals and prandial status on baseline diameter was not found, and because the postprandial FMD after curry even increased despite that the increase of baseline diameter should decrease FMD.

It is reported that a single consumption of a high antioxidant spice blend attenuated postprandial insulin and triglyceride responses and increased some plasma measures of antioxidant activities [33]. The results of the present study, however, showed that no interaction effects (i.e., the effects due to spices) on biochemical data between meals (control/curry) and prandial status (fasting/postprandial) were significant. In the previous study, the subjects were given a test meal with different composition, and with different kind of spices. Moreover, they collected blood samples at 30-min intervals for 3.5 h. These differences in experimental conditions may have affected the results.

We found that postprandial diastolic blood pressure decreased and postprandial heart rate increased significantly, but postprandial systolic blood pressure did not change significantly. Berry et al. reported that glucose solution with ground beef (i.e., solid food), when fed to human subjects, caused higher postprandial systolic blood pressure than glucose solution alone (i.e., liquid food) [34]. They also reported that diastolic blood pressure fell and heart rates increased, without significant (observable) effects from the ground beef [34]. Although the mechanisms were not fully understood, the results of our study in which participants consumed solid meal, agree with their findings.

There are some limitations in the current study. The first limitation is our small sample size (n = 14). Follow-up work using a larger sample size may be needed. The second limitation is that because of the unique flavor and color of spices used in the curry, the curry meals were readily distinguishable from the control meals by the subjects. Although we cannot exclude the possibility entirely that this feature of our study had some influence on our results, it is more likely that the presence of spices in the curry reduced the impact of the postprandial state on the endothelium after the meal.

## Conclusion

Curry consumption ameliorates postprandial endothelial dysfunction and may be beneficial for preventing cardiovascular events. Lifestyle-related diseases such as atherosclerosis and diabetes mellitus have become serious health problems in the modern world. Curry may be helpful in the fight against those lifestyle-related diseases.

## Abbreviations

FMD: Flow-mediated vasodilation; HDL-C: High-density lipoprotein-cholesterol; LDL-C: Low-density lipoprotein-cholesterol; hs-CRP: High-sensitivity C-reactive protein; MDA-LDL: Malondialdehyde-modified LDL; OGTT: Oral glucose tolerance test; Cr: Creatinine.

## Competing interests

This study was supported by House Foods Corporation. HN, SY and SS are employees of House Foods Corporation. NT, HS, and NM are employees of House Foods Group Inc. YH has no competing interest.

## Authors’ contribution

HN designed the research, performed measurements and statistical analysis, and wrote the manuscript. NT conducted the research. HS provided statistical consultation and critically revised the manuscript. NM provided much consultation about the study design and conducted the research. SY designed and prepared test meals. SS provided much consultation about the study design. YH provided much consultation about the study design, conducted the research, provided consultation about the discussion of the test results, and critically revised the manuscript. All authors read and approved the final manuscript.
